# Cutaneous vasculitis: Lessons from COVID-19 and COVID-19 vaccination

**DOI:** 10.3389/fmed.2022.1013846

**Published:** 2022-12-09

**Authors:** Alberto Corrà, Alice Verdelli, Elena Biancamaria Mariotti, Valentina Ruffo di Calabria, Lavinia Quintarelli, Cristina Aimo, Cord H. Sunderkötter, Marzia Caproni

**Affiliations:** ^1^Section of Dermatology, Department of Health Sciences, University of Florence, Florence, Italy; ^2^Immunopathology and Rare Skin Diseases Unit, Department of Health Sciences, Azienda USL Toscana Centro (ERN-SKIN), University of Florence, Florence, Italy; ^3^Department of Dermatology and Venereology, University Hospital Halle (Saale), Martin-Luther-University Halle-Wittenberg, Halle (Saale), Germany

**Keywords:** vasculitis, cutaneous vasculitis, COVID-19, leukocytoclastic vasculitis, IgA vasculitis, urticarial vasculitis, COVID-19 vaccines, vaccine-induced vasculitis

## Abstract

Cutaneous vasculitis (CV) is an inflammatory skin-limited vascular disease affecting the dermal and/or hypodermal vessel wall. From the pathogenetic point of view, idiopathic forms are described as well as the induction from various triggers, such as drugs, infections, and vaccines. Following SARS-CoV-2 pandemic outbreak, cases of CV induced by both COVID-19 and COVID-19 vaccinations have been reported in literature. The aim of our work was to collect multiple cases available in the literature and analyze the frequency of the different forms of induced vasculitis, as well as their histological and immunopathological features. Although rare, CV induced by Severe Acute Respiratory Syndrome Coronavirus 2 (SARS-CoV-2) and vaccines may provide interesting insights into the pathogenesis of these inflammatory processes that may in the future be useful to understand the mechanisms underlying cutaneous and systemic vasculitis.

## Introduction

The term vasculitis encompasses a wide and heterogeneous group of disorders with shared histopathological findings. It is a pathological process characterized by an inflammatory process affecting the vessel wall, both arterial and venous, of different sizes and of any body area (1). Inside the vessel wall, there is an infiltrate, which can create discontinuity of the wall itself with red blood cells leaking. One of the most successful attempts at proper classification of such condition has been proposed by the 2012 Chapel Hill consensus cVonference nomenclature of vasculitides (CHCC 2012) (2), which divides them according to the diameter of the affected vessel: Large Vessel Vasculitis and Medium Vessel Vasculitis, which in the skin can cause necrosis and ulceration and livaedo reticularis; Small Vessel Vasculitis, manifesting with purpura and vesiculo-bullous lesions.

Since the skin is one of the most affected organs in vasculitides, in 2018, a Dermatological Addendum has been suggested to further help the clinician in dealing with such conditions, improving the definition of some forms of cutaneous vasculitis (CV) and adding other dermatological relevance (3). Accordingly, CV may be a cutaneous manifestation of systemic vasculitis or a skin-limited or skin-dominant variant of systemic vasculitis, but when affecting only the skin in the absence of any other systemic involvement, the term single-organ vasculitis (SOV) should be used.

CV is mainly a small-vessel vasculitis affecting dermal and/or hypodermal capillaries and venules, which usually show histopathologic findings consistent with leukocytoclastic vasculitis, characterized by fibrinoid necrosis of vessel wall, erythrocyte extravasation, and neutrophilic infiltrate with degeneration known as leukocytoclasis with nuclear dust (karyorrhexis) (4). The immune infiltration may be mainly lymphocytic in lesions that appeared more than 48 h before. Direct immunofluorescence (DIF) of lesional skin is helpful in the diagnosis of CV, with maximum efficacy for the diagnosis of IgA vasculitis and lupus vasculitis. It can aid in the accurate diagnosis even when the histological changes are minimal (5–7). However, DIF positivity is strongly influenced by the timing of the biopsy (8).

Even though in more than half cases of CV it is impossible to assess the disease-inducing or promoting factor, it is well-known that the most common triggering factors are related to immunopathogenic mechanisms secondary to infections or drug intake (9, 10). Therefore, it is not surprising that since the beginning of the COVID-19 pandemic and after the introduction and administration of COVID-19 vaccines on a global scale, cases of COVID-19-associated and vaccine-associated CV have been reported (11–13).

When involving the skin, clinical manifestations of the COVID-19 infection show a great range of signs and symptoms (14). Five major classes of cutaneous manifestations in the setting of COVID-19 infection have been proposed by Tan et al. (15), e.g., pseudo-chilblains lesions, urticarial rash, vesicular (varicella-like) eruption, maculo-papular rash, and vaso-occlusive lesions. Several cases of both new onset and flares of CV have also been linked to COVID-19 and SARS-CoV-2 vaccination. However, they are not included in the aforementioned classification due to their low frequency (12, 16, 17).

Similarly, many heterogeneous cutaneous reactions to COVID-19 vaccination have been reported and classified by Shakoei et al. into the following major categories: local site reactions, type 1 (immediate) hypersensitivity reactions, type 4 (delayed) hypersensitivity reactions, autoimmune-mediated reactions, functional angiopathies, and reactivation of other viral conditions (18). In this classification, CV are classified among the auto immune-mediated reactions. Most of the cases reported occurred after the administration of messenger ribonucleic acid (mRNA)-based vaccines (19). In the literature, vaccine-associated CVs have been more frequently reported than CVs secondary to the COVID-19 infection. The number of persons that received at least one dose of the vaccine worldwide is larger when compared to that of the persons who contracted the infection. However, it is known that the vaccine reproduces only a small degree of adverse effects provoked by the natural infection of the immune system. Therefore, more vaccine-associated CVs are diagnosed and reported due to the greater attention that has been given by patients to all the side effects related to the COVID-19 vaccine.

In this review, we analyze and compare the current and most recent literature on clinical and immunohistopathologic features of CV induced by systemic SARS-CoV-2 infection and CV secondary to the SARS-CoV-2 vaccine, focusing on the possible underlying pathogenetic mechanisms.

## SARS-CoV-2 infection and cutaneous vasculitis

We collected clinicopathological features of a series of CV that occurred in association with the SARS-CoV-2 infection available in the literature (Table 1). Our search was restricted to cases with histological confirmation of leukocytoclastic vasculitis. Totally, 19 cases were included, mostly males (13/19) with variable age distribution ranging from 13 to 93 years with an average of 48.4 years. In three cases, the diagnosis was COVID-19-associated IgA vasculitis, while in five cases the patients had been diagnosed with COVID-19-associated urticarial vasculitis; finally, the other cases may be considered as cutaneous leukocytoclastic vasculitis associated with COVID-19, being not further classified according to the Dermatologic Addendum to the 2012 Revised International Chapel Hill Consensus Conference Nomenclature of Vasculitides (3). Regarding the clinical presentation, a comparison between the frequency of different types of lesions did not reveal feasible given the heterogeneity of their description. However, it is reasonable to consider palpable purpura as the main clinical manifestation, sometimes with necrotic features and hemorrhagic blistering. The most common sites affected were the lower limbs and trunk, as for the idiopathic forms of CV. The cases diagnosed with urticarial vasculitis showed slight clinical differences, since skin lesions were characterized by wheals or urticarial manifestations, associated with purpuric aspects. The edematous component of cutaneous lesions in COVID-19-associated urticarial vasculitis was appreciable at histological evaluation in 2 out of 5 cases, whose report mentioned dermal or endothelial swelling. The latency time between skin rash occurrence with SARS-CoV-2 infection is highly variable, ranging from concomitant signs appearing at the time of onset to more than 30 days after the first positive nasopharyngeal swab. The totality (3/3) of COVID-19-associated IgA vasculitis cases presented kidney involvement, but it is of interest that in two out of three cases, the direct immunofluorescence (DIF) performed on lesional skin resulted negative while positivity was seen in all three cases when performed on kidney biopsy. Although based on a few cases, our results are in accordance with Jedlowski *et al*., which published a case series of 10 subjects with COVID-19-associated systemic IgA vasculitis; in fact, authors found positive skin DIF in less than half of the series (40%) while kidney biopsies showed IgA deposition in all the cases. Moreover, it is of note that COVID-19-associated IgA vasculitis more commonly affects adults when compared to the classical form of IgA vasculitis in which 90% of cases occur in the pediatric population. In our series, one DIF resulted non-specifically positive for C3, while in nine cases, it was negative for all the reactants. No cases of cutaneous IgG/IgM vasculitis were diagnosed and in eight subjects DIF was not performed. Interestingly, three cases assessed the colocalization of SARS-CoV-2 in the vessel wall, finding positivity in 2/3 cases by the PCR technique. This may support the direct role of SARS-CoV-2 in the pathogenesis of cutaneous vasculitis and its tropism for a broad variety of human tissues.

**Table 1:** Clinical, histological, and immunological findings in patients with COVID-19-associated CV.

**Case no**	**Age**	**Sex**	**Comorbid**	**Time to infection**	**Clinical presentation**	**Histology**	**DIF**	**SARS-CoV-2 in dermal vessels**	**Ref**
1	93	M	CKD	8 days	purpuric macules and papules on legs, hands, and periumbilical area	Fibrin deposition,	Negative for IgG, IgA, IgM, C3	N/A	Capoferri et al. (45)
			PAD Hypertension			Obliteration of vessels			
						Extravasated red blood cells			
2	66	M	T2DM Hypertension CAD	15 days	Palpable purpuric papules with necrotic center	Fibrin extravasation in vascular structures	Negative for IgG, IgM, IgA, C3	N/A	Bay et al. (46)
						Inclusion bodies in endothelial cells			
					Maculo-papular lesions on legs and forearms				
						Perivascular neutrophil, lymphocyte infiltrate Leukocytoclasis in the dermis			
									
3	16	F	None	N/A	Edematous, maculopapular erythematous rash on extremities, abdomen, back, thighs and face	Neutrophilic vasculitis	Negative for IgG, IgM, IgA, C3	N/A	Gosnell et al. (47)
						Karyorrhectic debris			
						Focal degeneration of vessel wall			
						Rare intraluminal fibrin deposits			
						Micro-thrombi			
4	13	M	None	28 days	Petechial and purpuric rash on both feet and ankles	Superficial epidermal necrosis	Negative for IgG, IgM, IgA, C3	Positive (PCR)	Kumar et al. (48)
						Small-vessel neutrophilic vasculitis			
5	32	F	Crohn disease	14 days	Erythematous to violaceous macules and papules on lower extremities and dorsum of feet	Perivascular karyorrhectic material	Not performed	N/A	Nassani et al. (49)
						Stromal edema and purpura			
						Capillary ectasia			
						Thrombotic vasculopathy			
6	49	M	None	14 days	Palpable purpura on inferior limbs and abdomen	Hyperkeratosis	Not performed	N/A	Iraji et al. (50)
						Moderate neutrophilic infiltration			
						Extravasated red blood cells			
						Lymphocytes around dermal vessels			
7	70	M	None	N/A	Palpable petechiae on dorsal feet, thighs, abdomen	Leukocytoclastic vasculitis	Positive for IgA	N/A	Jedlowski et al. (51)
					Purpuric plaques				
8	27	M	None	N/A	Painful purpuric papules	Leukocytoclastic cutaneous vasculitis	Negative for IgG, IgM, IgA, C3	N/A	Gouveia et al. (52)
					Vesicobullous hemorrhagic lesions Necrotic lesions	Microthrombi			
9	43	M	Hypertension	N/A	Painful hemorrhagic bullae	Leukocytoclastic vessel vasculitis	Negative for IgG, IgM, IgA, C3	N/A	Kösters et al. (53)
					Necrotic lesions on trunk, arms, legs	Neutrophilic infiltration			
						Eosinophils and histiocytes			
10	29	M	None	28 days	Purple palpable papules	Heavy neutrophilic infiltrate in small vessel wall	Negative for IgG, IgA, IgM, C3	Positive (PCR)	Camprodon Gómez et al. (54)
					Necrotic lesions				
					Serohaematic blisters on abdomen, buttocks, lower legs, feet	Leucocytoclasis			
						Fibrinoid necrosis			
						Extravasation of red blood cells			
11	47	M	Hypertension, impaired glucose tolerance	18 days	Multiple, raised erythematous wheals, alone or in cluster, some with central purple Hyperpigmentation on head, trunk and upper arms	Orthokeratotic hyperkeratosis	Not performed	N/A	Skroza et al. (55)
						Spongiosis			
						Focal lymphocytic exocytosis			
						Perivascular neutrophilic infiltration			
									
						Vessel wall damage			
									
12	64	F	Hypertension, T2DM	Concomitant	Annular and polycyclic urticarial lesions with purpuric component on trunk and limbs	Dermal edema	Not performed	N/A	Nasiri et al. (56)
						Leukocytoclastic vasculitis			
13	59	M	N/A	35 days	Maculopapular purpuric exanthema on face, trunk, limbs	Perivascular neutrophilic infiltrate	Not performed	N/A	Caputo et al. (57)
						Leucocytoclasis			
						Red blood cell extravasation			
						Fibrinoid necrosis of vessel walls			
14	N/A	F	N/A	N/A	Painful erythematous patches on trunk, hips	Red blood cell extravasation	Not performed	N/A	de Perosanz-Lobo et al. (58)
					Purpura	Neutrophilic perivascular inflammation			
						Karyorrhexis			
15	N/A	M	N/A	N/A	Erythematous and edematous plaques with a purpuric center	Perivascular neutrophilic inflammation	Not performed	N/A	de Perosanz-Lobo et al. (58)
						Red blood cell extravasation			
						Endothelial swelling			
						Necrotic lesions			
						Fibrin deposition			
									
16	79	F	N/A	7 days	Purpuric macules and papules on legs	Fibrinoid necrosis of vessel walls	Positive for C3	Negative (PCR)	Dominguez-Santas et al. (59)
						Transmural infiltration by neutrophils			
						Karyorrhexis			
						Leukocytoclasia			
						Red blood cell extravasation			
17	83	F	Hypertension	30 days	Purpuric palpable papules and serohematic blisters on lower legs, feet, toes	Perivascular neutrophils	Not performed	Not performed	Mayor-Ibarguren et al. (60)
			TIA			Fibrins in vessel wall of the dermis			
			AF			Leukocytoclasia			
			CKD						
18	30	M	No	Concomitant	Painful purpuric rash	Leukocytoclastic vasculitis	Negative for IgA, IgG, IgM, C3	Not performed	Li et al. (61)
19	22	M	None	Concomitant	Palpable purpura with central vesicles on extremities, gluteal region, lower abdomen	Perivascular infiltrate of neutrophils, lymphocytes	Negative for IgG, IgM, IgA, C3	Not performed	Sandhu et al. (62)
						Red blood cell extravasation			
						Fibrinoid necrosis of vessel wall			

CKD, chronic kidney disease; PAD, peripheral artery disease; T2DM, type 2 diabetes mellitus; CAD, coronary artery disease; TIA, transient ischaemic attack; AF, atrial fibrillation.

## SARS-CoV-2 vaccination and cutaneous vasculitis

In the mini-series presented (Table 2), only patients with histological confirmation of leukocytoclastic vasculitis were included. Totally, 39 patients developed CV after the COVID-19 vaccine. Women were found to be more involved than men, counting 24 females vs. 15 males developing CV. The weighted average of the patients reported was of 53.2 years (range 22–94).

**Table 2:** Clinical, histological, and immunological findings in patients with COVID-19-vaccine associated CV.

**Case no**	**Age**	**Sex**	**Vaccine type**	**Vaccine name**	**Exclusion of SARS-CoV-2 infection**	**Comorbid**	**Temporal relation to the vaccine**	**Clinical characteristics of CV reported**	**Systemic involvement**	**DIF**	**References**
1	30	M	Adenoviral vector-based	Johnson-Johnson	Negative nasopharyngeal RT-PCR swab	None	17 days after the first dose	Painful hemorrhagic papules and vesicles on soles, shins, elbows	Mild proteinuria	Granular deposits of IgM, C3, and fibrin/fibrinogen in the walls of the dermal small vessels	Betetto L et al. (63)
									Hypocomplementemia		
									Cryoglobulinemia		
2	45	M	Inactivated vaccine	Sinopharm	Not mentioned	None	2 days after the first dose	Papular lesions on upper and lower limbs	Pruritus	Not performed	Shakoei et al. (18)
3	61	F	Adenoviral vector-based	Oxford-AstraZeneca	Negative nasopharyngeal RT-PCR swab	Hypertension	5 days after the first dose	Pruritic erythematous-purpuric macules involving the lower legs, feet, buttocks, axillae, abdomen	Myalgia	Not performed	Criado et al. (13)
									Fatigue		
4	52	M	m-RNA-based	Moderna	Not mentioned	Not mentioned	11 days after the second dose	Erythematous, non-pruritic petechial rash on lower limbs	Not reported	Not performed	Gázquez Aguilera et al. (11)
5	80	M	m-RNA-based	BioNTech/ Pfizer	Negative serologic investigations	Psoriasis	4 weeks after the second dose	Targetoid erythematous lesions	Fever	Negative for IgG, IgM, IgA, C3	Wollina et al. (19)
									Fatigue		
									General malaise		
								Necrotic lesions on legs Erythematous lesions on the soft palate			
						Hemochromatosis					
						Nodular goiter					
								Purpuric macules on fingers and palmar creases			
								Splinter hemorrhages on nails			
6	57	F	Adenoviral vector-based	Oxford-AstraZeneca	Not mentioned	Fibrocystic mastopathy	5 days after the second dose	Purpuric macules and papules on lower legs	Not reported	Linear and granular deposition of IgM within small vessels	Fiorillo et al. (64)
						Hypertension					
7	51	F	m-RNA-based	Moderna	No prior history of SARS-CoV2 infection	Sjögren syndrome Cryoglobulinemic vasculitis	3 weeks after the second dose	Palpable purpura and ulcers Lower extremities pitting edema	Acute kidney injury	Not performed	Vornicu et al. (65)
									Nephrotic syndrome		
8	59	F	m-RNA-based	BioNTech/ Pfizer	No prior history of SARS-CoV2 infection	Sjögren syndrome Cryoglobulinemic vasculitis	2 days after the first dose	Palpable purpura	Fatigue	Not performed	Vornicu et al. (65)
								Small cutaneous malleolar ulcers	Fever		
									Myalgias		
									Acute kidney injury Nephritic syndrome		
9	55	F	Adenoviral vector-based	Oxford-AstraZeneca	Negative RT-PCR	None	5 days after the first dose	Palpable purpura on lower limbs	Fever	Negative	Sandhu et al. (66)
									Myalgia		
									Wrist swelling		
10	48	M	Adenoviral vector-based	Oxford-AstraZeneca	Negative RT-PCR	Hypertension	2 days after the second dose	Palpable purpura on hands, forearms, gluteal region, lower limbs	Fever	Negative	Sandhu et al. (66)
									Myalgia		
11	46	F	m-RNA-based	BioNTech/ Pfizer	Not mentioned	Psoriasis	2 days after the first dose (1st flare), 2 days after the second dose (2nd flare)	Exacerbation of palpable purpuric papules lower legs (first flare)	Not reported	Not performed	Cohen et al. (67)
						PsA					
						Irritable bowel syndrome Leukocytoclastic vasculitis					
								Palpable purpuric papules on the lower legs, feet, upper extremities, lower back, and abdomen (second flare)			
											
12	83	F	m-RNA-based	BioNTech/ Pfizer	Not mentioned	None	5 days after the second dose	Palpable purpura with erythema and edema on lower extremities	Elevated levels of C-reactive protein, elevated sedimentation rate,	Deposition of fibrinogen around superficial blood vessels	Larson et al. (68)
									Rheumatoid factor		
									Hypocomplementemia		
									Cryoglobulinaemia		
13	57	F	m-RNA-based	Not mentioned	Not mentioned	Epilepsy Bipolar disorder Depression	7 days after the first dose	Erythematous confluent papules and plaques involving trunk, extremities	Not reported	Not performed	Bostan et al. (69)
14	46	F	Inactivated	Covaxin	Negative oro-nasopharyngeal RT-PCR swab	None	5 days after the first dose	Palpable purpura on legs	Arthralgia	Not performed	Kar et al. (44)
									Ankle swelling		
								Pitting edema on ankles			
15	47	M	m-RNA-based	BioNTech/Pfizer	Not mentioned	Intermittent abdominal pain	3 days after the first dose (first episode); 4 days after the second dose (flare)	Reddish spots in his ankles (first episode)	Elevated C-reactive protein	C3/C4 deposits	Gambichler et al. (70)
									Proteinuria		
								Purpuric papules on legs, forearms (second episode)	Decreased glomerular filtration rate		
16	59	F	m-RNA-based	Moderna	Not mentioned	Hypertension Hyperlipidemia	1 day after the second dose	Violaceous petechiae on legs, pelvis, abdomen, upper limbs	Intermittent abdominal pain	Not performed	Ireifej et al. (71)
									Elevated C-reactive protein		
						Prediabetes Obesity COVID-19 in April 2020					
17	57	F	Inactivated	Sinopharm	Not mentioned	None	5 days after the second dose	Purpuric papules with central blistering	Fatigue	Not performed	Azzazi et al. (39)
									Arthralgia		
								Necrotic lesions			
								Black eschars on legs			
								Palpable purpura on thighs, buttocks, abdomen, back, forearms			
18	94	M	m-RNA-based	Moderna	Not mentioned	AF	10 days after the second dose	Palpable purpura	Not reported	IgA immune deposits in the blood vessel walls	Grossman et al. (72)
						Aortic valve replacement					
						Hypothyroidism					
						Anemia					
19	76	M	m-RNA-based	BioNTech/ Pfizer	Not mentioned	Liver cirrhosis	12 days after the second dose	Pruritic purpuric macules on hands, feet, legs, thighs, abdomen	Bloody diarrhea	Not performed	Mücke et al. (73)
						Heart failure					
						Previous gastroesophageal junction cancer and prostate cancer					
20	65	M	m-RNA-based	BioNTech/ Pfizer	Not mentioned	T2DM	2 days after the third dose	Purpuric palpable lesions on legs	Not reported	Not performed	Dicks et al. (74)
						Hypertension					
21	50	M	m-RNA-based	BioNTech/ Pfizer	Not mentioned	None	2 days after the second dose	Rash on the legs	Not reported	IgA-dominant immune deposits in the blood vessel walls	Mohamed et al. (75)
22	40	F	m-RNA-based	BioNTech/ Pfizer	Not mentioned	Hashimoto's thyroiditis	20 days after second dose	Purpuric rash on gluteal region	Headache	Not performed	Hines et al. (76)
23	57	M	Adenoviral vector-based	Oxford-AstraZeneca	Not mentioned	Hypertension	14 days after the first dose	Purpura on lower limbs, abdomen, trunk, head	Not reported	Not performed	Cavalli G et al. (77)
24	57	F	Adenoviral vector-based	Oxford-AstraZeneca	Not mentioned	Hypertension	5 days after the first dose	Palpable purpura on buttocks, legs, arms	Not reported	Negative for IgG, IgM, IgA, C3	Guzmán-Pérez et al. (78)
						Hypothyroidism					
25	77	F	Adenoviral vector-based	Oxford-AstraZeneca	Not mentioned	None	10 days after the first dose	Palpable indurated purpuric papules	Not reported	Negative for IgG, IgM, IgA, C3	Shahrigharahkoshan et al. (79)
								Erythematous plaques and bullae on lower limbs, hands. Purpuric lesions on soft palate, tongue			
26	68	F	Adenoviral vector-based	Oxford-AstraZeneca	Not mentioned	None	7 days after the first dose	Erythematous to purpuric non-blanching macules on lower extremities	Not reported	Not performed	Jin et al. (80)
27	60	F	Adenoviral vector-based	Oxford-AstraZeneca	Not mentioned	Chronic liver disease	11 days after the second dose	Painful purpuric lesions on lower limbs	Not reported	IgA and IgM deposits on the walls of postcapillary vessels	Fritzen et al. (81)
						Portal hypertension					
						Polycythemia vera					
						Hypothyroidism					
						T2DM					
28	76	F	Adenoviral vector-based	Oxford-AstraZeneca	Not mentioned	None	7 days after the first dose	Maculopapular rash on lower extremities	Hematuria	Not performed	Sirufo MM et al. (43)
									Arthralgia		
29	46	F	Inactivated	Covaxin	Negative oropharyngeal RT-PCR swab	None	5 days after the first dose	Purpuric papules on legs	Arthralgia	Not performed	Kar et al. (44)
									Ankle swelling		
30	31	F	Inactivated	Covaxin	Negative oropharyngeal RT-PCR swab	None	4 days after the second dose	Palpable purpura on left leg	Not reported	Not performed	Kharkar et al. (82)
								Pitting edema			
31	77	M	Adenoviral vector-based	Sinovac	Negative nasopharyngeal RT-PCR swab	None	2 weeks after the third dose	Palpable violaceous patches	Gastrointestinal involvement (abdominal pain, stool tests on occult blood-positive)	Negative for IgG, IgM, IgA, C3	Oskay et al. (83)
								Bullous hemorrhagic lesions on lower limbs, hands			
32	33	M	Adenoviral vector-based	Not mentioned	Mildly symptomatic COVID-19 three months before	None	3 days after the first dose	Violaceous eruption	Not reported	IgA deposition within small vessel walls	Bostan et al. (84)
								Erythematous macules			
								Palpable papules on legs, forearms			
33	91	F	m-RNA-based	BioNTech/Pfizer	No evidence of acute SARS-CoV-2 infection	Dementia Hypertension T2DM	4 days after the third dose	Palpable purpuric lesions on lower limbs	Not reported	Not performed	Carrillo-Garcia et al. (37)
34	38	M	m-RNA-based	BioNTech/Pfizer	Not mentioned	None	4 days before the first dose	Purpuric-erythematous macules, papules, and plaques on lower limbs	Arthralgia	Not performed	Altun et al. (36)
35	52	M	m-RNA-based	Moderna	Not mentioned	Not mentioned	11 days after the second dose	Erythematous, non-pruritic rash on legs	Not reported	Not performed	Gázquez Aguilera et al. (11)
								Petechiae on lower limbs			
36	42	F	m-RNA-based	BioNTech/Pfizer	Not mentioned	Hypertension Obesity	4 days after injection (dose number non-specified)	Cutaneous eruption on lower limbs, gluteal area	Not reported	Not evaluable	Erler et al. (85)
37	22	F	m-RNA-based	BioNTech/Pfizer	Not mentioned	None	7 days after the second dose	Small, red, raised, itchy lesions on legs. Purpuric lesions on lower limbs	Not reported	Not performed	Ripalta Colia et al. (38)
38	23	F	Inactivated	Sinovac	Not mentioned	None	36 h after first dose	Non-blanchable erythematous plaques with purpura on extremities	None	C3 and fibrinogen deposition around blood vessel walls	Bencharattanapet al. (86)
39	26	F	Inactivated	Sinovac	Not mentioned	None	4 h after first dose	Non-blanchable purpuric purpura on extremities	None	IgM, C3, and IgA deposition	Bencharattanaphakhi et al. (86)

CKD, chronic kidney disease; PAD, peripheral artery disease; T2DM, type 2 diabetes mellitus; CAD, coronary artery disease; TIA, transient ischaemic attack; AF, atrial fibrillation.

**Figure 1:**
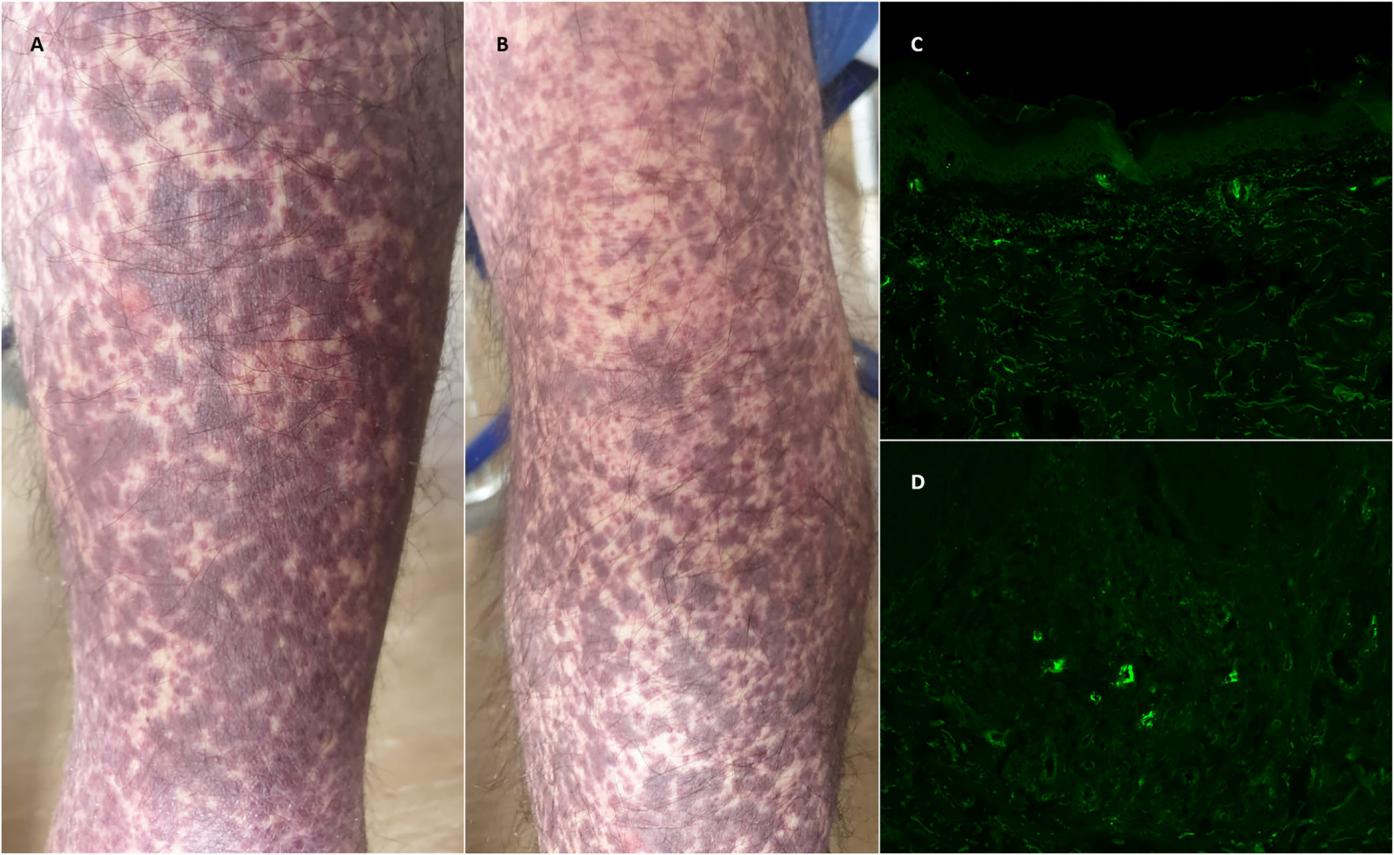
**(A,B)** Purpuric maculae and papules in the lower extremities in a patient with a recent anamnesis of COVID-19 vaccination. **(C,D)** Direct immunofluorescence performed on lesional skin, with evidence of perivascular deposition of C3. (c: 10% magnification, d: 20% magnification).

Clinically, purpuric papules or maculae in the lower extremities were the most commonly reported skin manifestation (Figure 1). DIF was not reported in 21 cases (53.8%) and in 5 cases (12.8%) it was negative. Features were heterogeneous in the remaining 13 cases, with 5 cases (12.8%) of IgA vasculitis and 3 cases (7.7%) of vasculitis with C3 deposition, and some isolated cases of IgM vasculitis with fibrinogen deposit.

Most of the reported cases (*n* = 19, 48.7%) were associated with mRNA vaccines; particularly, 13 patients underwent BNT162b2 [BioNTech/Pfizer] vaccines and five patients underwent mRNA-1273 [Moderna] vaccines. In one case, the commercial name of the vaccine was not reported. Eleven cases (28.2%) of CV were associated with adenoviral vector-based vaccines, of whom 10 were with ChAdOx1 nCoV-19 [Oxford-AstraZeneca] and one was with Ad26.Cov2.S [Johnson & Johnson].

Among the nine cases (23.1%) associated with inactivated vaccines, only one was not named, three cases were found after the administration of both Covaxin and Sinovac, and two cases after Sinopharm administration.

Nineteen patients (48.7%) developed CV after the first dose of the vaccine, while 16 (41%) after the second dose; only 3 (7.7%) cases were reported to occur after the third dose of the vaccine injection. In one case (2.6%), the dose number was non-specified.

## Discussion

Our review reported the main aspects of both CVs induced by COVID-19 infection and vaccines. Only leukocytoclastic vasculitis was included, and DIF pattern was also analyzed. Unfortunately, in many of the reported cases, DIF was not conducted, while some cases were negative. Its evaluation is extremely important in defining the type of CV and DIF positivity may raise the suspicion of systemic disease, providing useful prognostic information where histology alone cannot. Therefore, DIF should be always performed especially on early lesions because immune deposits may disappear in lesions that occurred more than 48 h before.

To date, the exact pathogenetic mechanisms underlying COVID-19-associated CV have not been fully understood. Since its outbreak in 2019, COVID-19 had spread all over the world causing a global pandemic affecting more than 500 million people and at least 6 million deaths (20). The enveloped RNA virus called Severe Acute Respiratory Syndrome Coronavirus 2 (SARS-CoV-2) is the etiologic agent, which primarily affects the respiratory tract leading to general symptoms like fever, fatigue, anosmia, and dysgeusia, while respiratory symptoms are variable in severity ranging from cough and rhinorrhea to dyspnea, pneumonia, or acute respiratory distress syndrome. However, evidence about the involvement of other organs and systems is increasing; in fact, knowledge about the neurological, gastrointestinal, and ocular manifestations of SARS-CoV-2 infection is deepening (21, 22). Similarly, cutaneous signs of COVID-19 are continuously reported and attempts at classifications are already available in the literature, together with the first prevalence estimations in which dermatologic manifestations would place between 1.8 and 20.4% of the COVID-19 patients (23, 24). In particular, several works identified clusters of skin manifestations that are suggestive of skin vascular damage, namely chilblain-like lesions, acral ischemia, acral vasculitis, livedo reticularis, livedo racemosa, purpuric “vasculitic” rash, or petechial eruptions (25–27). While a definitive nomenclature is justifiably actually lacking, considering the novelty of these entities, it is well known that SARS-CoV-2 features a markable tropism for endothelial cells. The first hypothesis of vascular damage provoked by the novel coronavirus was provided from autoptic studies showing platelet-fibrin thrombi in lung blood vessels in patients who died of severe COVID-19 (28), advancing the evidence of coagulopathy as a main pathogenetic mechanism of single- or multiorgan damage induced by SARS-CoV-2. Indeed, the term “immunothrombosis” is now used to refer to the typical pattern of lung damage resulting from massive viral-induced inflammation, which leads to the activation of the endothelium and triggers intravascular coagulation. Similar mechanisms may be responsible for skin manifestations reflecting vascular dysfunction or true vasculitis, since it was demonstrated that ACE2 is expressed in the skin basal cell layer, dermal vessels endothelium, eccrine glands, and subcutaneous fat tissue and act as a receptor for SARS-CoV-2 Spike protein binding (29). Viral uptake precludes the ACE2-dependent protective action of angiotensin 1–7 and results in oxidative stress, inflammatory cytokine production, and vasoconstriction (30, 31). Endotheliitis following virus internalization enhances endothelial injury, thrombogenesis, and immune recruitment, while the cytokine storm typical of severe cases may additionally boost the same mechanism in multiple anatomical districts (32). Moreover, sustained activation of the complement system causes microvascular injury and a procoagulant state triggered by the deposition of complement component C4d and colocalization of SARS-CoV-2 Spike protein in dermal vessels (33). All these mechanisms contribute to the inflammatory dermal microenvironment, which may be the subject of the innate and adaptive immune cell recruitment leading to the extension of inflammatory process toward the vessel wall, causing vasculitis. Another proposed pathogenetic mechanism may involve an autoimmune response targeting vessel wall components following a break of tolerance or molecular mimicry with SARS-CoV-2 proteins (34). Furthermore, CV was described in the context of Kawasaki-like syndrome, a generalized inflammatory disease affecting mainly infants for which the term “multisystem inflammatory syndrome in children (MIS-C) has been coined. However, the specificity of skin vasculitis in the setting of MIS-C still remains unclear, also due to the less frequency of skin biopsies performed in children.

All vaccines authorized for use by the U.S. *Food and Drug Administration* (FDA) and the European Agency for the Evaluation of Medicinal Products (EMEA) have been thoroughly studied and found to be safe and effective in preventing severe COVID-19 cases (35). However, as globally millions of people have now been vaccinated, with increasing frequency, vaccination-related diseases have been observed (36), including CV.

Almost all the available COVID-19 vaccines have been associated with CV, e.g., mRNA vaccines (Pfizer BioNTech), mRNA-1273 (Moderna), adenoviral vector-based vaccines (ChAdOx1 nCoV-19; Oxford-AstraZeneca), and inactivated vaccines (Covaxin, Sinovac). Correlations between vaccination and the subsequent appearance of several types of vasculitis have been also described in the literature with vaccines against influenza, hepatitis B, serogroup B meningococcus, hepatitis A, Human Papilloma Virus (HPV) and with Bacillus of Calmette-Guérin (BCG) (37).

An important criterion guiding the assessment of causality is the temporal relationship between immunization and the side event: for drug- and vaccine-induced vasculitis it is considered to be in the range of 1–6 weeks (38). Most of the cases were self-limiting skin forms without systemic involvement, solved spontaneously or after systemic treatment.

The link between vasculitis and vaccination from a pathogenetic point of view is not clear but may involve an immune complex and antibodies deposition in the blood vessel walls (39). Recently, cytoplasmatic granular positivity for SARS-CoV-2 Spike protein was found in some skin specimens of infection-related CV (40). The vaccine proteins are structurally analogous to the wild viral antigens and could induce a pro-inflammatory cascade similar to that caused by the viral protein. Thus, vaccine antigens may activate B/T cells and cause antibody formation with subsequent immune complex deposition in small-caliber vessels. Along with this, Baiu et al. demonstrated the role of Th1 response and suggested that interferon-gamma is critically required for the initiation of vascular inflammation (41). Then, the whole-virion inactivated SARS-CoV-2 vaccine induces primarily a Th1-biased response, which could lead to the induction of an inflammatory response in the vessel wall (42). An open issue for patients who developed such adverse events following COVID-19 vaccination is whether the booster dose should be administered or not. In fact, repeating the administration could potentially cause more severe immunologic reactions (43). However, cutaneous small-vessel vasculitis secondary to infections, drugs, and vaccines is reported to have a less protracted course when compared to primary vasculitis. Therefore, this should not be a deterrent to the use of the COVID-19 vaccine, which is the most effective weapon to curb the pandemic (44).

## Conclusion

Although rarely, CV has been reported in both SARS-CoV-2 -infected and SARS-CoV-2-vaccinated patients. In many cases, these were self-limiting skin forms without systemic involvement, solved spontaneously or after systemic treatment. Studies on this topic are however important to better understand the pathogenetic mechanisms underlying their origin.

With the evolution of the infection and with the finding of less aggressive SARS-CoV-2 variants, it will be necessary to follow the patients who will develop a CV, to better define their characteristics, and possibly understand which variants are more associated with the development of CV. Moreover, the epidemiological trend of COVID-19 infection and the need to protect especially the fragile population made it necessary to start a vaccination campaign with a fourth additional dose. Therefore, careful monitoring of these patients is essential to identify the presence of CV and to make a correct diagnosis, based not only on histological examination but also on DIF, essential to better define the characteristics of SARS-CoV-2 and vaccine-related CV.

## Author contributions

AV, CHS, and MC contributed to conception and design of the study. EM organized the database of cases collected. AC, EM, VR, and AV wrote the first draft of the manuscript. LQ and CA wrote sections of the manuscript. All authors contributed to manuscript revision, read, and approved the submitted version.
